# Transmission Dynamics of Imported Vaccine-Origin PRRSV-2 within and between Commercial Swine Integrations in Hungary

**DOI:** 10.3390/ani13193080

**Published:** 2023-10-02

**Authors:** Szilvia Jakab, Krisztián Bányai, Krisztina Bali, Imre Nemes, Ádám Bálint, István Szabó

**Affiliations:** 1 HUN-REN Veterinary Medical Research Institute, Hungária krt. 21., H-1143 Budapest, Hungary; jakab.szilvia@vmri.hu (S.J.); bali.krisztina@vmri.hu (K.B.); 2 National Laboratory for Infectious Animal Diseases, Antimicrobial Resistance, Veterinary Public Health and Food Chain Safety, Hungária krt. 21., H-1143 Budapest, Hungary; 3 Department of Pharmacology and Toxicology, University of Veterinary Medicine, István utca. 2., H-1078 Budapest, Hungary; 4 National PRRS Eradication Committee, Keleti Károly utca. 24., H-1024 Budapest, Hungary; nemesi@nebih.gov.hu; 5 Veterinary Diagnostic Directorate, National Food Chain Safety Office, H-1143 Budapest, Hungary; balintad@nebih.gov.hu

**Keywords:** NA-type PRRSV, molecular epidemiology, virus transmission, next-generation sequencing, single nucleotide variation, Ingelvac PRRS MLV, Hungary

## Abstract

**Simple Summary:**

Two recent transmission chains of PRRSV-2-associated cases were documented in this study using field epidemiological and molecular genetic tools. The investigation highlighted the risks associated with the free movement of livestock in the European Union. To minimize this risk of re-infection of PRRS-free herds with PRRSV through animal imports, it is recommended that pigs are transported directly from the exporting holdings without the involvement of transit stations. Alternatively, the transit stations could be converted so that pigs in transit avoid contact with each other, thus preventing exposure to PRRSV infection.

**Abstract:**

This study reports on the molecular epidemiology of Ingelvac-PRRS-MLV-associated cases in Hungary for the period 2020–2021. Field epidemiology investigations led the experts to conclude that imported pigs, which were shipped through transit stations in Denmark, introduced the vaccine virus. The movement of fatteners and the neglect of disease control measures contributed to the spread of the virus to PRRS-free pig holdings in the vicinity. Deep sequencing was performed to genetically characterize the genes coding for the virion antigens (i.e., ORF2 through ORF7). The study isolates exhibited a range of 0.1 to 1.8% nucleotide sequence divergence from the Ingelvac PRRS MLV and identified numerous polymorphic sites (up to 57 sites) along the amplified 3.2 kilo base pair genomic region. Our findings confirm that some PRRSV-2 vaccine strains can accumulate very high number of point mutations within a short period in immunologically naive pig herds.

## 1. Introduction

Porcine reproductive and respiratory syndrome (PRRS) is amongst the most significant infectious diseases constantly inducing great economic losses for the global swine production. The typical manifestations of the syndrome are reproductive disorder in sows and respiratory disease in piglets [1]. The increased mortality of prenatal, pre-weaned and young pigs as well as reduced growth rate have the most relevant economic impact [2,3]. The causative agent of PRRS is an enveloped, single-stranded RNA virus (PRRSV) belonging to the *Arteriviridae* family [4]. Two, distantly related PRRSV species are distinguished, such as *Betaarterivirus suid 1* and *Betaarterivirus suid 2* (former PRRSV-1 or EU-type and PRRSV-2 or NA-type, respectively) [5].

Recognized by the competent committee of the European Union, Hungary launched an eradication program (2014–) in order to reduce the initially high prevalence of PRRSV and to alleviate the associated economic burden [6]. By 2022, all pig herds became free of wild-type PRRSVs, and the legislation continues to support the maintenance of an absolute free status for pig holdings (including disease-free status from vaccine-origin PRRSVs) [7]. The extensive monitoring conducted throughout the years of disease elimination revealed that the circulating EU-type Hungarian PRRSV strains were genetically diverse, whereas NA-type PRRSVs were scarcely identified [8,9]. In fact, all NA-type viruses detected after 2014 were classified as derivatives of a live, attenuated vaccine virus [9]. The vaccine virus was developed from a wild-type virulent strain, VR2332 (https://bi-animalhealth.com/swine/products/flex/ingelvac-prrs-mlv (accessed on 29 September 2023.)). The vaccine (Ingelvac PRRS MLV or RespPRRS MLV) is registered in nine countries within the EU, such as Denmark, Belgium, Germany, Lithuania, Luxembourg, Netherlands, Poland, Portugal and Spain (https://www.ema.europa.eu/en/documents/referral/modified-live-porcine-respiratory-reproducti-article-35-referral-annex-i-ii-iii_en.pdf (accessed on 29 September 2023)). In addition, Slovakia permits the use of this vaccine for immunization on a case-by-case basis. Identification of vaccine-origin PRRSV-2 strains has been published from the EU [10,11,12,13,14], and the policies concerning the registration of the PRRSV-2 vaccines are likely to reflect the actual epidemiological situation in the respective countries; yet, updated surveillance data are not regularly published. Although the Ingelvac PRRS MLV is not authorized in Hungary, the introduction of the vaccine virus is plausible, for example, through illegal import of vaccines (e.g., as happened in 2008, I. Szabó, unpublished data) or with imported, freshly vaccinated piglets.

Denmark, where the Ingelvac PRRS MLV is regularly used, is a major source of pre-fatteners for Hungary, therefore it is essential that the Hungarian authorities are aware of the export procedures. Under Danish legislation, Hungary is a red zone country, therefore exported pigs must be loaded onto Hungarian trucks at EU-certified collecting stations, not directly at the sellers’ farm. Given that the transit stations place pigs from various farms in shared air space, even a PRRS-free shipment could be infected with various PRRSVs before entering Hungary [15].

In this study, we present the molecular epidemiological aspects of two PRRS outbreaks associated with the Ingelvac PRRS MLV. We wish to draw attention to the mode of infection, which is related to the commercial logistics of immunized fattening pigs in a foreign country.

## 2. Materials and Methods

### 2.1. Epidemiological Investigation

Our epidemiological investigations in relation to the accumulation of NA-type PRRSV infections involved seven large-scale fattening and breeding farms (Farm A to Farm G) during 2020–2021 (Table 1). The inspection of official departments included the checking of import licenses, the documents accompanying the shipments (TRACES certificate), and the results of diagnostics. At the same time, information was collected on the movement of transport vehicles (live animals and carcasses, forages, breeding material, etc.) passing through other establishments or slaughterhouses linked to the farms concerned. Additionally, the personnel entering the examined pig farms were recorded.

### 2.2. PRRSV Diagnostics

Serum or buccal swab samples were collected from imported prefattener stocks, from pigs showing clinical symptoms and from pigs involved in regulatory PRRSV monitoring (pregnant gilts, sows and fatteners). In general, the number of samples taken for diagnostics is calculated based on 95% confidence at 10% prevalence values [16]. The viral RNA was isolated with the QIAmp Viral RNA Mini Kit (Qiagen, Hilden, Germany) and tested for PRRSV with RT-qPCR using the Virotype PRRSV RT-PCR Kit (Qiagen, Hilden, Germany) according to the manufacturer’s instructions. Sequencing of ORF5 or ORF7 was performed on randomly selected samples exhibiting low Ct values obtained in the diagnostic RT-qPCR assay (for details see [17]). Sequencing of these amplified regions using the Sanger method was carried out on an ABI PRISM 3100 automatic sequencer. Antibody detection was performed using the PRRS Universal ELISA Kit (Ingenasa, Madrid, Spain) according to the manufacturer’s instructions.

Concerning the imported fattening stocks, the measures initiated by the eradication program in accordance with the law are as follows. The entering prefatteners are quarantined for 60 days, and diagnostic tests are conducted (PRRSV RNA and antibody detection) 48 h within their arrival. At the end of the quarantine, the whole workflow is repeated. In addition to the monitoring of imported fattening stocks, regular and mandatory surveillance is performed every six months as required by the law [18].

### 2.3. Analysis of the ORF2-7 Region

The standard diagnostic procedures were supplemented with amplification and next-generation sequencing (NGS) of the structural ORFs, ORF2 to ORF7. In brief, the viral RNA was freshly re-isolated from the remaining specimens with the QIAmp Viral RNA Mini Kit (Qiagen, Hilden, Germany). SuperScript^TM^ III Reverse Transcriptase (Invitrogen, Waltham, MA, USA) and an anchored poly(dT) primer (5′-TTTTTTTTTTTTTTTTTAATTWCG-3′) were used for cDNA synthesis. Amplification of the ORF2-7 region was performed with the Phusion DNA polymerase (Thermo Scientific, Waltham, MA, USA). The final volume of PCR mixture was 20 µL, in 1× HF Buffer with 3% DMSO, 0.5 µL of each primer (10 µM), and 1 µL of cDNA template. The degenerate primer pair was as follows: 5′-CGKGCGCGCCAGRAAGGGAAAATTTA-3′ and 5′-GCACARTRTCAATCAGTGCCATTCAC-3′. The parameters of the PCR program were as follows: initial denaturation at 98 °C for 30 s, then 35 cycles of denaturation at 98 °C for 10 s, annealing at 66 °C for 30 s and extension at 72 °C for 2 min; the final elongation at 72 °C lasted for 10 min. After analysing the PCR product on 1% agarose gel, the bands were excised and purified with Gel/PCR DNA Fragments Extraction Kit (Geneaid, New Taipei City, Taiwan). For library preparation, we used the Nextera XT DNA Library Preparation Kit and the Nextera XT Index Kit v2 (Illumina, San Diego, CA, USA). Sequencing was performed on an Illumina^®^ NextSeq 500 sequencer, as described previously [19]. GenBank accession numbers of the study strains are as follows: OR143097-OR143102.

### 2.4. Sequence Analysis

The whole ORF2-7 region was assembled from the NGS short reads via reference mapping to the VR2332 prototype genome. Variant calling (minimum variant frequency of 10%) was performed in Geneious 9.1.8 software (Biomatters, Inc., Auckland, New Zealand). The final consensus sequences were aligned with selected lineage-specific sequences using the MAFT algorithm as implemented in Geneious 9.1.8. Pairwise sequence identities were generated in the MEGA software (version 10.1.8) [20]. Phylogenetic reconstruction based on maximum likelihood was performed with MEGA software (version 10.1.8) (model HKY + G, 1000 bootstrap).

## 3. Results

### 3.1. Transmission Chains

Preceding the incidents, all affected pig herds in this study possessed a PRRS-free status registered by the Hungarian Veterinary Authorities. However, from autumn 2020 to summer 2021, two distinct transmission chains of the Ingelvac PRRS MLV vaccine virus were identified. In this section, we describe the timeline of sampling and diagnostic test results followed by the traditional and molecular epidemiologic findings.

#### 3.1.1. Transmission Chain I

All identified cases in the transmission chain I were connected to the collecting station “P” in Denmark.

Farm A: Several shipments arrived from a foreign, certified PRRS-free farm to Farm A through the same collecting station in Denmark (“P”) on each week in October and twice in the first two weeks of November 2020. In mid-October, 30 samples were subjected to laboratory tests from the first two stocks; however, that was several days after the mandatory 48 h. All samples had a positive PCR result, 20 samples also gave positive ELISA and a single PRRS EU strain (Porcilis PRRS vaccine strain) was detected via ORF5 sequencing. Neither relevant blood test nor ELISA were performed for the subsequent stocks.

Farm B: Routine laboratory tests of serum samples (n = 33) from Farm B were positive for PRRS (39% PCR positive and 100% seropositive) on 18 November 2020. The ORF7 sequence determined for a single sample (sample ID.: 64196) showed the closest genetic similarity to Ingelvac PRRS MLV.

Farm C: On 15 and 20 April 2021, sera (n = 11) and buccal swabs (n = 8) were collected from sows, respectively, which manifested mild clinical symptoms such as loss of appetite, fever and snorting. PRRS infection was confirmed with positive ELISA (100%) and PCR tests (100%). The ORF7 and ORF5 sequences (sample ID.: 18601, sow, buccal swab) identified the Ingelvac PRRS MLV as the causative PRRSV strain. Subsequently, the authorities ordered herd closure and monitoring of all age groups, and serum samples (n = 190) were gathered on 21 April. The obtained seropositivity and positive PCR rate (found in the cohorts of 28- and 60-day old pigs) for the corresponding herd were 74% and 7.9%, respectively. The ORF7 and ORF5 sequences (samples ID.: 19001, pig, serum) were classified as Ingelvac PRRS MLV.

Farm D: Fattening stocks were continuously placed on Farm D from Farm C. On 21 April 2021, routine serological tests showed 100% seropositivity and 16.7% of serum samples (n = 30) were PCR-positive in case of the fatteners (90–180 days old) settled in January and February of 2021. Sequencing of the ORF7 (samples ID.: 19702) resulted in the detection of Ingelvac PRRS MLV. Furthermore, seropositive animals were found among pigs settled in April 2021.

Farm E: Fattening stocks were continuously placed on Farm E from Farm C. On 23 April 2021, routine serological tests from serum samples (n = 160) showed almost 100% seropositivity and PCR positivity (1.9%) of the examined herd of Farm E, and Ingelvac PRRS MLV was identified after sequencing the ORF5 gene (sample ID.: 19601).

#### 3.1.2. Transmission Chain II

All identified cases in the transmission chain II were connected to the transit station “T” in Denmark.

Farm F: Prefatteners purchased from a foreign-certified PRRS-free farm were imported through the transit station “T” in Denmark to Farm F on 26 May 2021. The mandatory laboratory tests from serum samples (n = 29) performed immediately after unloading showed high rates of positivity with PCR (100%) and ELISA (13.8%). The following diagnostic tests (7, 14 and 27 days after the settlement) confirmed the infection with PRRSV, and the Ingelvac PRRS MLV was identified via sequencing of the ORF7 (sample ID.: 27392).

Farm G: Prefatteners were ordered from a foreign-certified PRRS-free farm and transported through the collecting station “T” in Denmark to Farm G on 4 May 2021. Serology testing (n = 29) was conducted within 48 h after the arrival, which gave negative test results. At the end of the quarantine (7 July 2021); however, the herd displayed positivity with PCR (100%) and ELISA (100%) tests, and the pathogen was classified as the Ingelvac PRRS MLV based on ORF7 sequencing.

**Figure 1:**
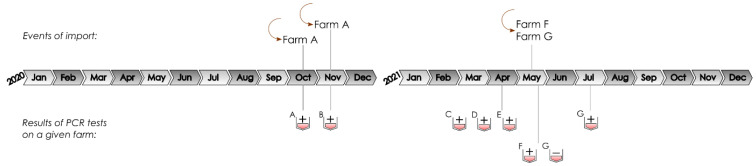
Schematic presentation of pig import events and the results of PRRSV PCR tests.

The events of pig import and the results of the PRRSV PCR tests conducted for this study are summarized on a timeline figure (Figure 1).

### 3.2. Epidemiological Investigation

**Figure 2:**
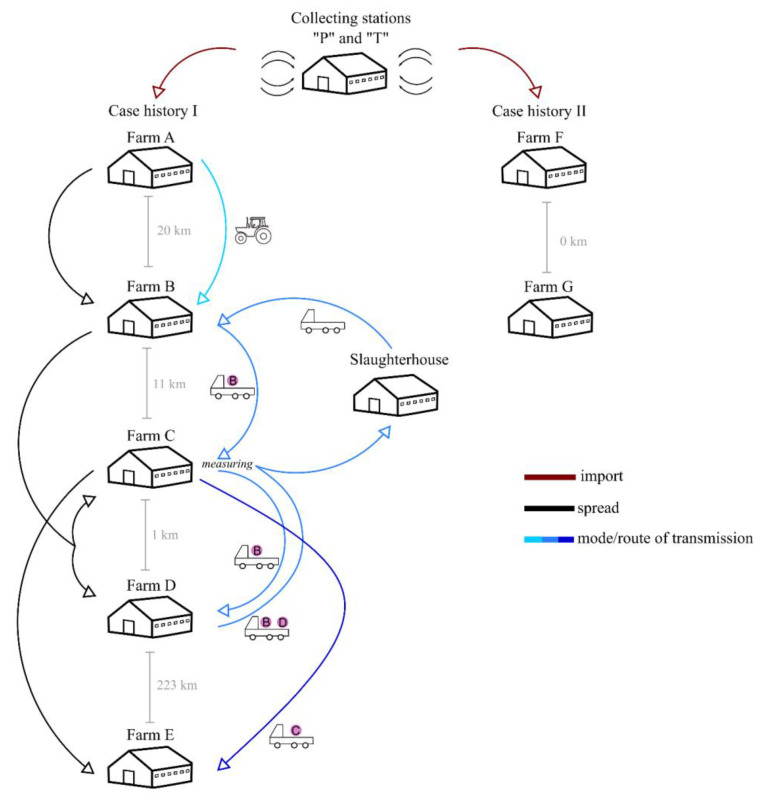
The most likely transmission routes of the Ingelvac PRRS MLV vaccine strain determined during field epidemiology investigation. The arrows indicate the movement of pigs and the possible routes of vaccine strain transmission.

Epidemiological investigation was performed to explore the most likely transmission route of the Ingelvac PRRS MLV vaccine strain among affected herds (Figure 2).

In the case series linked to collecting station “P” in Denmark, five large-scale farming units were involved, and Farm A was identified as the initial piece of the transmission chain. Pigs imported from Denmark through collecting station “P” to Farm A (October and November, 2020) could be infected recently with the Ingelvac PRRS MLV vaccine virus. From some stocks of Farm A, we identified the Porcilis PRRS vaccine strain, an EU-type vaccine that is regularly used for immunization in Denmark. The PRRSV-2 vaccine virus itself could not be detected at Farm A, in part, as a result of omitting routine virological testing of some imported stocks. Concerning the next steps in virus transmission, field investigation suggested that a manure suction vehicle, which regularly operated between Farm A and Farm B, was responsible for the introduction of PRRSV from Farm A to Farm B. Importantly, Farm B had no direct transport or any other commercial relationships with collecting station “P” or Farm A that could have explained the introduction of vaccine virus to this farm. Next, the authorities recognized that the route of infection to other farms could be epidemiologically linked to the management of fattener transport to the slaughterhouses or the pig movement between consecutive phases of production. An apparent issue was that weighing equipment was not available at Farm D; therefore, the transport vehicle owned by a local slaughterhouse was used to transport the pigs at to Farm C, where measuring equipment was available. Considering this practice, infection of Farms C and D with the vaccine strain may have occurred when the vehicle of the slaughterhouse collected infected pigs from Farm B on 17 February 2021, then the animals were weighed at Farm C. Next, the vehicle picked up fatteners at Farm D and returned to Farm C to weigh the pigs of Farm D. At last, the vehicle completed its journey at the slaughterhouse. Throughout the process of loading, the staff members had multiple contact events with infected animals, including the transport vehicle and many objects located at the farm that could have readily spread the vaccine virus. Another plausible explanation is that only Farm C was infected this way, and later, fatteners from Farm C were placed in Farm D, which caused the emergence of vaccine-associated PRRS on Farm D. In case of Farm E, the transportation of already infected prefatteners from Farm C to Farm E introduced the vaccine-origin PRRSV.

In the case series linked to collecting station “T” in Denmark, two large-scale fattening holdings were exposed to the vaccine virus. A shipment to Farm F from Denmark very likely carried the PRRS vaccine strain, as diagnostic tests upon arrival identified severe PRRS infection in imported piglets. Farms F and G are two neighbouring establishments, with a fence separating them on the side boundary. The field investigation established that the fence and the negligible distance between the pens of the two farms could unlikely prevent the spread of vaccine virus to the PRRS-free herd of Farm G.

### 3.3. Molecular Investigations

Considering only the ORF5 and ORF7 regions, which were sequenced during the diagnostic workflow, the nucleotide (nt) and amino acid (aa) sequence identity among samples were 96.4% to 100% (nt) and 97.5% to 100% (as) for the ORF5, and 97.6% to 100% (nt) and 99.2% to 100% (as) for the ORF7. Both the ORF5 and ORF7 sequences shared high similarities to the Ingelvac PRRS MLV vaccine strain (98–100% and 98.1–100%), respectively. Thus, the sequence data obtained for ORF5/ORF7 uncovered the vaccine origin of strains in disease etiology; however, additional data were needed to support the field epidemiology observations. The genomic region encoding the structural proteins was amplified and sequenced from samples collected at Farms B, C, D, E and F. Unfortunately, some imported stocks having arrived at Farm A were not sampled, and amplification of the ORF2-7 region from samples collected at Farm G failed.

**Figure 3:**
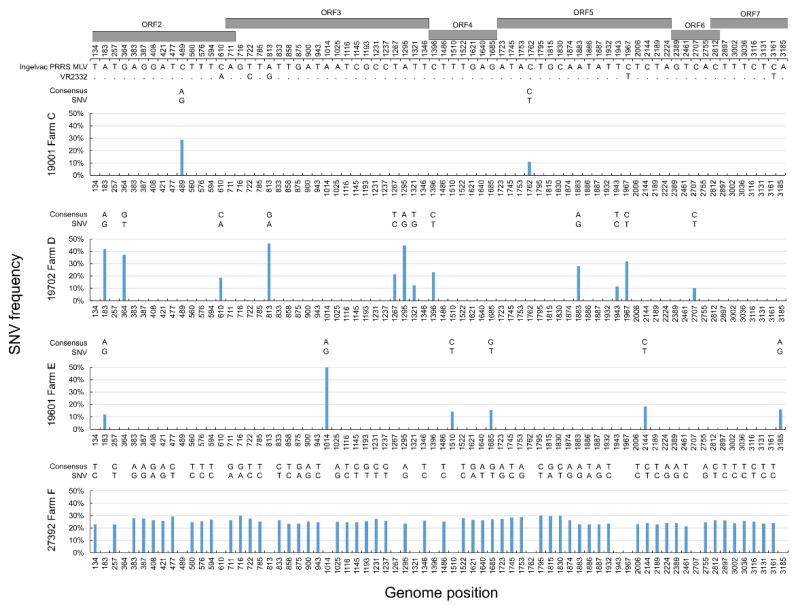
Distribution of SNV sites in the ORF2-7 region of study strains and their comparison to the consensus sequences of Ingelvac PRRS MLV and VR2332.

The ORF2-7 region was uniformly 3188 base pair long, neither indel mutations nor recombination events were detected. Pairwise nt identities of the obtained ORF2-7 consensus sequences fell between 97.5% and 100%, and, when compared to the Ingelvac PRRS MLV vaccine strain, the identities ranged between 98.2% and 99.9% (Table 2). By analysing the NGS runs, we found a total of 72 SNV sites in four samples (19001, 19702, 19601 and 27392) under the criterion of a minimum variant frequency of 10%. The other two samples (64196 and 18601) showed no sequence variation in the amplified genomic region. The frequency of SNVs, analysed at an average sequencing depth of 8976–22052X, ranged from 10.2% to 50.8% (Figure 3). The position of SNVs varied considerably among strains. Between the Ingelvac PRRS MLV and the VR2332 strain, the following nt positions were identified as being different along the ORF2-7 region: 610, 722, 813, 1967 and 3161. Only, two and one SNV variants identical with the VR2332 were detected in samples 19702 and 27392, respectively, at positions 610, 772 and 1967.

**Figure 4:**
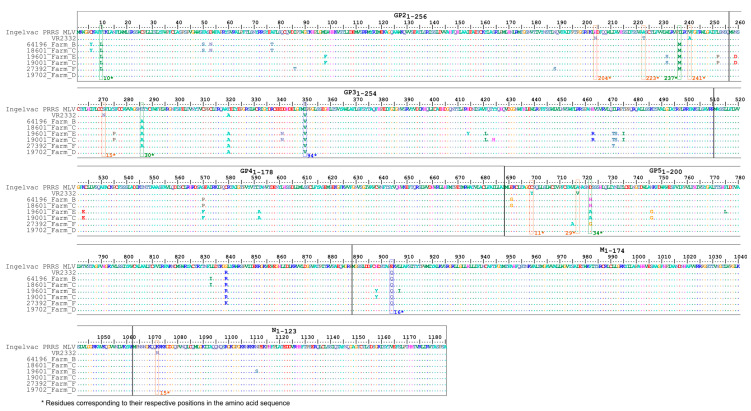
Concatenated amino acid alignment of the reference and Hungarian PRRSV-2 strains. Colour code: green, unique changes that differed from both reference strains; orange, shared residues with the Ingelvac PRRS MLV; blue, shared residues with the wild-type virus strain, VR2332.

**Figure 5:**
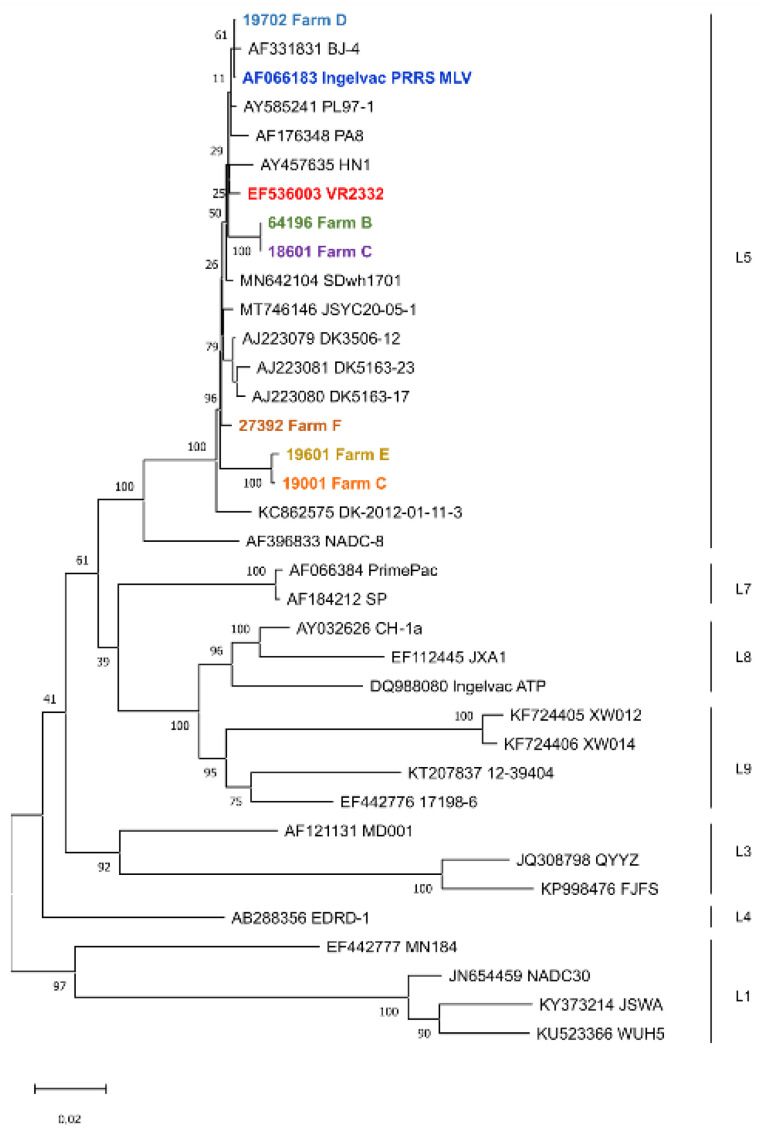
Maximum-likelihood-based phylogenetic tree based on the ORF2-7 region of the genome. The study strains and the reference strains, Lelystad and VR-2332, are highlighted with different colours. For a better resolution of phylogenetic relationships among strains, additional sequences from the GenBank were also used. The lineage specificities are shown on the right.

When analysing the aa identity values in the amplified structural protein region, the study strains were found to share 95.3–100% identities with the Ingelvac PRRS MLV vaccine strain, with GP3 being the most different region. In particular, the number of amino acid positions showing substitutions within GP2, GP3, GP4, GP5, M and N of the Hungarian isolates compared to the vaccine strain were the following: 11/256 (4.3%), 13/254 (5.1%), 3/178 (1.7%), 7/200 (3.5%), 3/174 (1.8%) and 1/123 (0.8%), respectively. As shown in Figure 4, the Hungarian study strains shared some unique aa residues (such as two (F^10^ → L^10^, I^237^ → M^237^), one (T^30^ → A^30^) and one (D^34^ → H/A/G^34^) aa residues in the GP2, GP3 and GP5, respectively) that were shared by the majority of study strains (with the exception of sample originating from Farm D) but differed from both the vaccine strain and its wild-type parental strain. We observed several aa residues that were identical to the Ingelvac PRRS MLV within GP2, GP3, GP5 and N, at three (H^204^, A^223^ and V^241^), one (I^15^), two (C^11^, A^29^) and one (K^10^) positions, respectively (Figure 5). On the contrary, only two aa residues situated in the GP3 (I^94^ → V^94^) and M (E^16^ → Q^16^), except 19702 from Farm D, were equal to the VR2332, respectively (Figure 5).

Phylogenetic analysis was performed to connect genetic data to the epidemiologic observations. The ORF2-7-based phylogenetic tree confirmed the relationship among the Hungarian strains involved in the two PRRSV-2-associated infection chains, and these strains clustered with the Ingelvac PRRS MLV vaccine virus, the parental VR2332 strain and other vaccine-related strains from different countries (Figure 5). Sequences from Farms B and C, likewise C and E, formed two distinct clusters while the sequence originated from Farm D clustered with the Ingelvac PRRS MLV.

## 4. Discussion

Eradication of PRRSV from the pig population of Hungary is nearing completion. As the number of PRRS-free herds increases, it is a fundamental obligation for the authorities to maintain the PRRS-free status. A key measure in these efforts is the continuous epidemiologic surveillance and the elimination of all PRRS infections, including those resulting from vaccination with live virus. Thus, awareness of the major epidemiological risks greatly contributes to the effective control of PRRS.

The international regulation by the World Organisation for Animal Health (WOAH) requires the verification of the occurrence of PRRS infection according to the following criteria: (i) isolation of PRRSV from pig, except vaccine strains; (ii) detection of PRRSV’s antigen or RNA, which is not the consequence of vaccination; (iii) detection of the vaccine PRRSV strain’s specific antigen or RNA from non-vaccinated pig; (iv) detection of antibody against PRRSV, which is not the consequence of vaccination (https://www.woah.org/ (accessed on 09 September 2023)). The Hungarian law added a further criterion declaring that fattening units must be free of both wild-type and vaccine-origin PRRSV [6].

From the perspectives of epidemiological judgment and regulation, the vaccine-origin PRRSV strains are considered less harmful, and their impact on economic losses is negligible when compared to infection with wild-type variants. Apparently, this is in accordance with the requirements of vaccine use. However, vaccine viruses introduced without vaccination into a non-vaccinated pig population may serve as a possible source of infection as can be seen in the case of wild-type strains [21,22,23].

In this study, the dispersal routes and the transmission modes of the NA-type Ingelvac PRRS MLV vaccine origin strain was sought in Hungary via epidemiological investigation and virus genetic analysis. Our investigations led to the conclusion that imported pig stocks that temporarily stayed at collecting stations in Denmark were the primary source of infection. Although the number of samples whose sequence data were available for analysis was limited, our virus genetic data helped clarify the epizootiology of vaccine-origin PRRS infection in the affected swine herds. Analysis of the ORF2-7 region of all available NA type PRRSV study strains showed close genetic relationship to the Ingelvac PRRS MLV strain. Moreover, the deduced amino acid sequences uncovered greater number of shared residues with the vaccine strain than with the wild-type VR2232 strain.

One of the epidemic cases presented here underlines the responsibility of the importer (Farm A), as without the proper examination of imported pigs, a previously scarce, identified live vaccine strain—the Ingelvac PRRS MLV—was introduced into five Hungarian PRRS-free pig herds. The Ingelvac-PRRS-MLV-related variants detected on Farm B (sample 64196) and one obtained from sows on Farm C (sample 18601) showed 99% nt identity to the vaccine strain and clustered together in the phylogenetic tree. This finding implies that these sequences may represent an earlier infection in the transmission route as the strain barely differed from the original vaccine strain. Furthermore, it confirms the spread of PRRS by the slaughterhouse’s vehicle operating between these two pig farms during the period of infection. At Farm C, another sequence (sample 19001) was identified that originated from a piglet; this sample shared 97.6% nucleotide identity with sample 18601 (from a sow), suggesting a greater level of virus divergence during the infection cycle among swine of different ages, a finding that has been reported previously [24]. This latter sequence from Farm C and sample 19601 from Farm E showed 98.3% and 98.2% nt identity to the vaccine virus, respectively. The genetic difference suggested that the circulating PRRSV strains in these herds could have been the most genetically distant viruses from the initially imported vaccine virus. A recent paper reported that sequences gathered during outbreaks could accumulate high number of mutations [24]. In Denmark, the Ingelvac PRRS MLV derivative sequences showed a divergence of up to 6% [12], whereas in the USA, less than 5% divergence to the vaccine was observed in most vaccine-related strains [25,26]. Additionally, our previous study also supports the possibility of this level of genetic variation among vaccine-derived PRRSV-2 strains [9]. Results from the phylogenetic analysis were consistent with the theory that PRRSV was introduced to Farm E by the settled fatteners from Farm C. Sample 19702 from Farm D shared 99.9% nucleotide identity with the Ingelvac PRRS MLV strain. We hypothesize that at the very beginning of the vaccine virus transmission, infected fatteners from Farm B were transported to the slaughterhouse. Afterwards, the virus spread to Farm D when the slaughterhouse’s vehicle was loaded for weighing of fatteners of Farm D. In this case, this particular virus variant showed less variation compared to the original vaccine virus suggesting that it may have undergone less animal-to-animal passage in different herds. Collectively, tracking the routes of the transmission chain we revealed that within six months (from November 2020 to April 2021), a newly introduced vaccine-origin PRRSV strain was able to spread to several different establishments (in this case four) and could leap great geographic distances that may jeopardize the PRRS-free status of herds in parts of the country.

There are two main factors contributing to the spread of PRRSV-2 in Hungary, based on our previous and current observations: the initial introduction by importing infected pigs and subsequent circulation among different herds [9]. Of note, due to the strict measures implemented in this country, newly introduced strains are eradicated rapidly. However, legal measures to control PRRSV could be hindered if, for example, external biosecurity measures are occasionally neglected in farm management or in case of shared equipment and vehicles or inadequate location of facilities.

Epidemiological investigation convincingly identified the causative virus beyond the accumulation of cases and provided relevant clues concerning the vaccine virus transmission; however, some uncertainties remained. Throughout the study period the detected vaccine-origin PRRSV-2 sequences showed 0.1% to 1.8% nt distance from the Ingelvac PRRS MLV along the ORF2-7 region. The diversity among all strains identified in this study was even greater and reached up to 2.5% nt divergence with some genomic region being even more divergent (ORF5, up to 3.6%). Thus, the observed sequence diversity in the sample set was somewhat greater than expected within this relatively short period of time. However, this is not a unique finding, given that the maximum genome-level sequence divergence detected so far within a year was as high as 6.4% [12]. We speculated that deep sequencing may help define whether a mixture of closely related vaccine strain-origin variants co-circulate because of the viral quasispecies features. From this viewpoint, a limitation of the study was that sampling for sequencing was randomly carried out, and the population structure of vaccine virus derivates could not be determined in simultaneously sampled animals. Despite this limitation, the strategy of choosing deep sequencing uncovered some details concerning the intra-host evolution of vaccine strain from independent herds. We chose a 10% cut-off value to define a minor variant in the sample as a possible marker for the quasispecies structure. Four out of six samples showed evidence of intra-host viral diversity, whereas two samples were genetically homogenous. In samples with multiple co-evolving virus, the SNV sites ranged from 2 to 57 along the 3188 bp fragment coding for the major virion antigens. Seeing this divergence and the ratio of the minor and the major sequence variants, we can conclude that the SNV structure cannot be directly related to the possible mutation rates derived from the chemistry of PCR or the sequencing technology. Indeed, the data obtained in this study, show that the Ingelvac PRRS MLV vaccine strain may become fairly diverse, raising questions concerning the rate of evolution and selection of dominant variants in individual animals as well as in affected herds. An important finding was that the distribution of SNVs did not show a tendency to revert to the exact form of the wild-type parental strain, VR2332, in any samples including those identified in pigs showing clinical manifestations. Different virus genomic evolution was also reported before when analysing the population structure of a single Ingelvac-PRRS-MLV-related strain [27]. We found no correlation between the number of SNVs and the sampling location within the transmission chain either; therefore, it is not clear whether the number of minor genomic variants increases over time in pigs reared in successive facilities. A more thoughtful sampling process in similar situations might help understand if the observed sequence divergence among samples is driven by random effects or if there are yet unidentified driving forces that act on the microevolution of PRRSV genomes.

## 5. Conclusions

In this paper, two distinct transmission chains of PRRSV-2-associated cases are reported, where epidemiologic investigation highlighted the risks associated with free movement of livestock in the EU. Although PRRS is still a major source of economic losses for the swine industry, it is not a notifiable disease in EU countries and no legislation was implemented for the regulation of pig trade on an EU standard. As reported previously, it is proven that imported prefatteners are a major threat to maintain the PRRS-free status of herds [18]. The ratification of swine stock import solely from PRRS-free pig farms restricted the entry of severely infected pigs to Hungary. Subsequently, an additional critical problem became the focus of attention. The export practice, such as the utilization of collecting stations, is considered as a primary animal health risk for Hungary, particularly in case of PRRSV-2 strains that are not endemic in Hungary. Temporary cohabitation of pigs from different herds at the same facility could be a main source of infection, as the pigs are in different health status and may be in different stages of infection. To minimize the risk of PRRSV infection through import and thereby reduce further damage to farmers, it is recommended that pigs are transported directly from the exporting holdings without the involvement of transit stations. Alternatively, these stations could be designed so that pigs from different herds do not come into contact and thus are unable to transmit infections to each other. In parallel, another critical task to reduce the risk of PRRSV spread is to improve biosecurity and regulatory measures within pig holdings in Hungary.

## Figures and Tables

**Table 1:** Farms involved in the transmission chain of Ingelvac PRRS MLV.

Farms	Farm Size and Type	Origin of Pigs	Other Information
Farm A	4000-seat large-scale fattening farm, continuously supplied	Prefatteners are purchased from Denmark	Situated in the same county
Farm B	600-seat large-scale breeding farm (Hungarian Large White x Hungarian Landrace local hybrid), farrow-to-finish type establishment		
Farm C	large-scale breeding farm (1820 sow, Danbred hybrid), farrow-to-finish type establishment	Parts of prefatteners are transported to a separate farm or other integrators	
Farm D	2700-seat large-scale fattening farm, continuously supplied	Prefatteners are from Farm C	
Farm E	9000-seat large-scale fattening farm, continuously supplied	Prefatteners are from Farm C	-
Farm F	2000-seat large-scale fattening farm continuously supplied	Prefatteners are purchased from Denmark	-
Farm G	2600-seat large-scale fattening farm continuously supplied	Prefatteners are purchased from Denmark	-

**Table 2:** Percent pairwise nucleotide identities among study strains and selected reference strains.

Strains	VR2332	Ingelvac PRRS MLV	64196 Farm_B	18601 Farm_C	19001 Farm_C	19702 Farm_D	19601 Farm_E	27392 Farm_F
VR2332								
Ingelvac PRRS MLV	99.6							
64196_Farm_B	98.8	99						
18601_Farm_C	98.8	99	100					
19001_Farm_C	98.1	98.3	97.6	97.6				
19702_Farm_D	99.6	99.9	99	99	98.3			
19601_Farm_E	98.1	98.2	97.5	97.5	99.7	98.2		
27392_Farm_F	99.3	99.4	98.8	98.8	98.3	99.5	98.2	

## Data Availability

Sequence data were deposited in GenBank (OR143097-OR143102). Additional data are available from Dr. István Szabó via the e-mail address shown on the title page.
